# Characterization and analysis of an infectious bronchitis virus strain isolated from southern China in 2013

**DOI:** 10.1186/s12985-016-0497-3

**Published:** 2016-03-09

**Authors:** Gang Xu, Xiao-yu Liu, Ye Zhao, Yang Chen, Jing Zhao, Guo-zhong Zhang

**Affiliations:** Key Laboratory of Animal Epidemiology and Zoonosis, Ministry of Agriculture, College of Veterinary Medicine, China Agricultural University, No. 2 Yuanmingyuan West Road, Haidian, Beijing, 100193 People’s Republic of China

**Keywords:** Infectious bronchitis virus, China, Sequence analysis, Phylogenetic analysis, Recombination

## Abstract

**Background:**

Infectious bronchitis is a severe disease caused by infectious bronchitis virus (IBV) that affects fowl flocks worldwide. The understanding of the mechanisms involved in IBV evolution and variation would provide important theoretical basis for prevention and control of the disease in the future.

**Methods:**

IBV strain GD was isolated from southern China in 2013 and the complete genome sequencing and phylogenetic analysis were performed.

**Results:**

The genome of approximately 27,680 nt comprised six genes, with insertions and mutations in most of the structural genes. The S1 gene showed the highest identity to strain TW2575/98 isolated in Taiwan, and was distantly related to the H120 vaccine strain. Phylogenetic analysis showed that the S1 gene of strain GD was also related to that of TW-type strains. Recombination analysis indicated that strain GD was a chimera whose putative parental strains belonged to the QX- and TW-type subgroups.

**Conclusions:**

An increasing number of TW-type strains have been isolated from China in recent years, which is in agreement with our findings, suggesting the emergence and increased prevalence of new TW-type strains in southern China.

## Background

Infectious bronchitis virus (IBV), a member of genus *Gammacoronavirus*, subfamily *Coronavirinae*, family *Coronaviridae*, affects the performance of both meat-type and egg-laying birds, causing tremendous economic losses in the poultry industry worldwide [1, 2].

IBV is an enveloped, positive-sense, non-segmented, single-stranded RNA virion,approximately 27.6 kb in length that contains 5ʹ and 3ʹ untranslated regions [3, 4]. The genome of IBV contains at least 10 open reading frames (ORFs) as follows: 5ʹ-1a-1b-S (S1, S2)-3a-3b-3c (E)-M-5a-5b-N-Poly (A)-3ʹ [5]. The two overlapping ORFs of 1a and 1b at the 5ʹ end are encoded by Gene 1, which constitutes approximately two-thirds of the genome, and are translated as the large polyprotein 1ab that is associated with RNA replication and transcription. The other regions of the genome encode four main structural proteins (the glycosylation spike glycoprotein (S), the envelope protein (E), the membrane protein (M), and the nucleocapsid protein (N)), as well as two accessory genes, 3 and 5, which express accessory proteins 3a and 3b, and 5a and 5b, respectively [6]. The spike protein of IBV is cleaved into S1 and S2 glycoproteins. The S1 subunit is the major target of neutralizing antibodies and carries serotype-specific antigenic determinants. The C-terminal portion of the S2 protein intercalates in the virus envelope and assists S1 protein anchoring in the membrane [7]. Both M and E proteins are membrane-associated proteins required for the formation of virus-like particles and virus budding [8]. N protein is associated with the RNA genome and forms the ribonucleoprotein [2].

An increasing number of new serotypes or variants of IBV caused by frequent gene mutation have become the major challenge of the prevention and control of IB [9]. Multiple IBV serotypes have been identified worldwide and the number of emergent IBV antigenic variants is increasing [7, 10, 11]. Meanwhile, the little cross-protection between different variants and vaccine has led to the outbreaks of infectious bronchitis in vaccinated chicken flocks [11–13]. Genomic recombination has been shown to occur at high frequency in coronaviruses, particularly IBV [14–17], also potentially leading to outbreaks of disease. All of these findings suggest the importance in the investigation of new and emerging IBV isolates.

In China, the outbreaks of infectious bronchitis in vaccinated and non-vaccinated flocks has led to severe economic losses in recent years, highlighting the need for a comprehensive study in the epidemic status of IBV serotypes. Therefore, we monitored the clinical manifestations of infectious bronchitis among Chinese chicken flocks and isolated a number of strains affecting flocks from different regions. One such strain, strain GD, was isolated from the flock in Guangdong Province and the complete genome was sequenced. Phylogenetic analysis was performed by comparing the genome sequence of strain GD with that of other IBV strains, including reference strains, vaccine strains, and other Chinese isolates reported recently. Recombination sites were analyzed to determine whether strain GD was a chimera. Our results provide insight into the current distribution of IBV genotypes across China and the molecular characteristics of prevalent strains. These findings may provide important theoretical basis and practical guidance for the effective vaccine strategies in prevention and control of IBV.

## Results

### Sequence analysis of the strain GD genome

The full-length genome sequence of IBV isolate GD was obtained and was found to be about 27,680 nucleotides (nt) in length. The genome comprised six genes and 10 ORFs in the following organization: 5ʹ-1ab-S-3a-3b-E-M-5a-5b-N-3ʹ. Gene 1 was 19,904 nt in length and consisted of two overlapping ORFs: 1a and 1b. Gene 2 encodes the S protein and contained 3, 498 nt. S gene was found to be cleaved into two subunits, S1 and S2, which were 1, 620 and 1, 878 nt in length, respectively, encoding the S1 protein of 540 aa and the S2 protein of 626 aa. The cleavage site on the spike protein was R-R-F-R-R. Gene 3 encoded non-structural proteins 3a and 3b, and structural protein E, which were 174, 189, and 327 nt in length, respectively, and the size of animo acid were 58, 63 and 109 aa. Gene 4 encoded the M protein of 226 aa and contained 678 nt. Gene 5 encoded two proteins, 5a and 5b, which were 66 aa and 83 aa, translated by 198 and 249 nt, respectively. Gene 6 was 1230 nt and encoded the N protein of 410 aa.

### Sequence comparisons with other IBV genomes

Comparison of the full-length genome sequences showed that the nt identities between strain GD and other IBV strains were ranged from 84.6 to 94.4 %. Strain GD had the highest nt sequence identity (94.4 %) to strain YX10 (GenBank accession number JX840411) isolated from southern China and the lowest nt sequence identity (84.6 %) to strain Georgia 1998, a vaccine strain. The nt identity between strain GD and the H120 vaccine strain was 86.5 % (Table 1).

**Table 1:** Sequence identity of the genome and individual genes of strain GD to other IBV strains

Strain	Genome	S1	S2	3a	3b	E	M	5a	5b	N
YN	90.0	82.7 (83.4)	93.4 (94.7)	92.0 (87.9)	75.1 (87.1)	90.2 (91.7)	94.8 (97.3)	95.5 (97.0)	92.4 (90.4)	87.6 (91.5)
YX10	94.4	78.3 (80.1)	94.3 (94.9)	90.2 (82.8)	82.0 (58.1)	92.7 (91.7)	94.7 (97.3)	82.8 (78.8)	92.4 (86.7)	88.8 (90.7)
DY07	94.1	78.1 (79.5)	94.5 (95.0)	90.2 (82.8)	79.2 (58.1)	93.0 (92.7)	94.5 (97.3)	89.9 (89.4)	97.2 (94.0)	93.8 (96.3)
Sczy3	94.1	78.2 (79.9)	94.4 (95.0)	89.7 (81.0)	75.0 (58.1)	93.0 (92.7)	94.4 (96.9)	84.3 (81.8)	92.8 (90.4)	93.1 (95.1)
CK/CH/SD/121220	94.2	79.1 (78.8)	94.3 (94.9)	90.2 (81.0)	76.3 (61.3)	93.3 (92.7)	94.1 (97.3)	91.4 (87.9)	94.4 (92.8)	93.9 (96.3)
SDIB821/2012	94.3	78.7 (79.5)	94.4 (95.0)	90.2 (81.0)	76.8 (58.1)	92.7 (91.7)	94.0 (96.9)	84.8 (81.8)	95.6 (92.8)	95.0 (96.3)
CQ04-1	93.3	82.4 (83.2)	94.4 (95.5)	92.0 (87.9)	76.6 (91.9)	91.7 (90.8)	93.7 (93.4)	93.9 (92.4)	92.0 (89.2)	87.3 (90.0)
SC021202	90.1	82.9 (83.7)	93.4 (94.9)	92.0 (87.9)	82.1 (83.9)	89.9 (91.7)	93.4 (97.8)	96.0 (97.0)	92.0 (89.2)	87.7 (91.5)
SAIBK	89.3	82.3 (83.8)	93.3 (94.7)	92.0 (87.9)	82.1 (80.6)	89.0 (90.8)	93.4 (96.9)	85.9 (83.3)	92.0 (89.2)	87.7 (90.5)
A2	90.1	82.4 (82.2)	91.6 (94.1)	87.9 (86.2)	87.9 (59.7)	89.9 (91.7)	93.1 (93.4)	87.4 (84.8)	98.0 (96.4)	92.1 (93.4)
TW2575/98	88.0	98.3 (96.8)	91.2 (93.9)	87.9 (82.8)	77.3 (58.1)	88.1 (90.8)	92.8 (94.7)	81.8 (81.8)	91.6 (88.0)	88.7 (91.0)
LX4	90.0	78.7 (80.0)	89.7 (93.9)	90.8 (82.8)	82.6 (56.5)	90.2 (92.7)	92.8 (93.8)	87.4 (86.4)	96.0 (96.4)	92.4 (96.1)
4/91	86.2	77.3 (76.7)	85.3 (89.2)	87.4 (82.8)	87.4 (64.5)	85.2 (87.7)	90.7 (92.5)	86.9 (78.8)	92.8 (90.4)	90.7 (93.9)
FL18228	86.7	82.0 (82.3)	86.2 (89.6)	84.5 (82.8)	82.1 (59.7)	87.0 (84.0)	90.6 (94.2)	81.8 (77.3)	93.2 (92.8)	90.0 (93.9)
Ck/CH/LDL/101212	86.3	81.2 (81.5)	85.5 (88.9)	83.9 (81.0)	83.9 (66.1)	85.3 (82.6)	90.6 (93.8)	83.8 (80.3)	92.0 (90.4)	93.5 (94.9)
Ck/CH/LNM/091017	86.5	81.2 (81.5)	85.4 (88.9)	83.9 (81.0)	78.8 (66.1)	85.3 (84.3)	90.6 (93.8)	83.3 (78.8)	92.0 (90.4)	89.4 (93.4)
H120	86.5	81.2 (81.5)	85.4 (88.6)	83.9 (81.0)	76.1 (66.1)	85.3 (82.6)	90.6 (93.8)	83.3 (78.8)	92.0 (90.4)	89.3 (93.2)
H52	86.4	81.2 (81.4)	85.4 (88.4)	83.3 (79.3)	92.9 (67.7)	85.6 (85.3)	90.3 (93.8)	82.8 (78.8)	90.4 (86.7)	88.7 (92.9)
Conn46 1996.	86.7	81.7 (82.0)	86.2 (90.0)	85.1 (84.5)	76.8 (67.7)	86.3 (83.2)	90.2 (93.3)	82.3 (77.3)	93.2 (92.8)	90.1 (93.9)
M41	86.2	81.6 (81.0)	85.5 (88.3)	85.1 (79.3)	90.8 (67.7)	85.9 (85.3)	90.1 (94.2)	79.8 (74.2)	91.2 (88.0)	88.6 (92.2)
Georgia 1998 Vaccine	84.6	61.6 (49.8)	74.7 (74.8)	85.6 (82.8)	81.5 (58.1)	86.9 (87.2)	90.1 (93.8)	81.8 (75.8)	93.2 (92.8)	90.0 (93.7)
Beaudette	86.5	81.7 (81.7)	85.2 (87.9)	86.2 (84.5)	86.2 (67.7)	85.9 (84.4)	90.1 (92.9)	83.3 (77.3)	92.8 (92.8)	89.2 (91.5)
Gray	86.7	78.7 (78.8)	86.2 (86.9)	83.9 (81.0)	82.0 (66.1)	85.7 (83.2)	90.1 (65.2)	83.8 (80.3)	92.0 (90.4)	88.9 (84.1)
Ck/CH/LHLJ/100902	86.5	81.6 (81.0)	85.5 (88.6)	85.1 (79.3)	85.1 (67.7)	86.2 (86.2)	90.0 (93.8)	80.3 (74.2)	90.8 (88.0)	88.4 (92.0)
Holte	86.6	79.2 (76.3)	85.3 (85.1)	81.6 (79.3)	77.2 (56.5)	86.6 (84.1)	90.0 (91.9)	83.8 (80.3)	92.0 (90.4)	88.5 (91.2)
Ck/CH/LHLJ/07VII	86.3	80.4 (79.3)	85.5 (88.4)	81.9 (71.9)	81.9 (67.7)	85.9 (85.3)	89.8 (93.4)	80.3 (74.2)	90.8 (88.0)	88.8 (92.7)
BJ	89.2	78.1 (79.1)	85.3 (88.9)	89.1 (86.2)	89.1 (66.1)	85.9 (86.2)	89.8 (91.6)	82.8 (83.3)	89.4 (86.6)	92.5 (94.1)
Delaware072	84.7	62.0 (51.1)	74.7 (67.9)	85.1 (81.0)	81.5 (67.7)	85.6 (83.5)	89.5 (93.8)	81.3 (77.3)	92.4 (91.6)	89.0 (91.2)
Ck/CH/LHB/100801	87.5	87.7 (85.7)	92.0 (94.5)	86.2 (87.9)	86.2 (61.3)	87.2 (88.1)	89.5 (91.6)	83.3 (81.8)	91.2 (90.4)	87.4 (89.5)
Arkansas Vaccine	86.5	78.3 (79.0)	86.2 (88.9)	87.4 (82.8)	87.4 (66.1)	86.0 (82.2)	89.4 (92.5)	82.3 (77.3)	92.4 (90.4)	89.7 (93.2)
KM91	87.5	83.7 (84.2)	92.3 (94.4)	82.2 (67.2)	78.3 (87.1)	89.6 (89.0)	88.9 (91.2)	80.8 (74.2)	92.0 (91.6)	90.2 (93.7)
ITA/90254/2005	87.9	78.8 (79.7)	91.2 (94.4)	86.8 (84.5)	77.3 (64.5)	86.5 (85.3)	88.8 (92.0)	87.4 (83.3)	90.0 (86.7)	91.0 (93.7)
SNU8067	86.8	77.2 (77.1)	91.5 (94.1)	82.2 (79.3)	89.1 (61.3)	88.0 (86.1)	88.6 (90.3)	80.8 (74.2)	91.2 (88.0)	89.3 (93.9)
GX-NN09032	91.7	66.8 (60.2)	74.8 (73.8)	83.9 (77.6)	82.0 (71.0)	85.9 (82.6)	88.2 (89.4)	90.9 (87.9)	91.2 (86.7)	87.3 (90.5)

Nucleotide (amino acid) sequence identity (%) are shown

### Sequence comparisons between gene segments

**Fig. 1:**
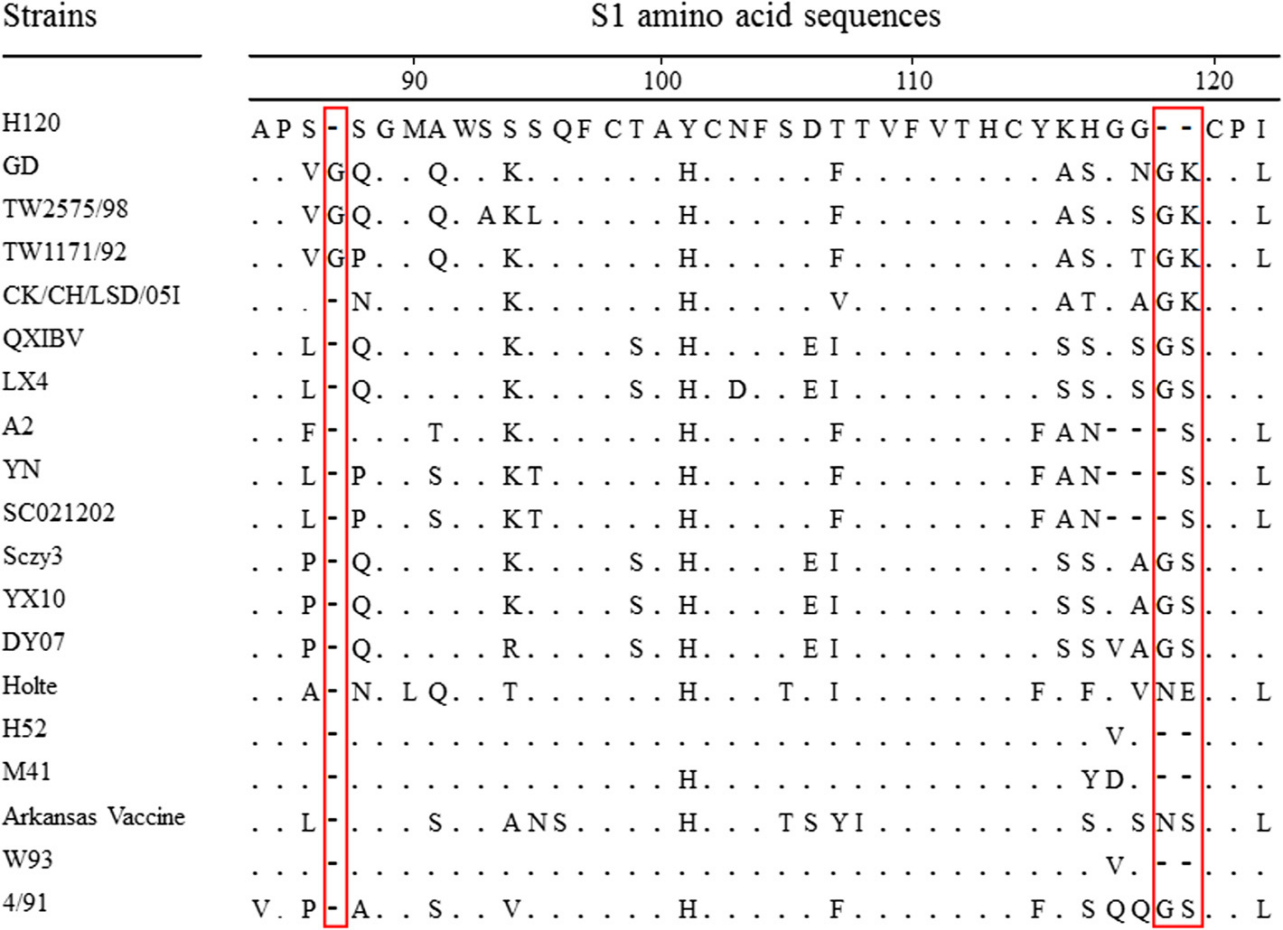
Sequence alignment of S1 amino acids sequence of GD strain and major reference IBV strains with H120. A *dot* indicates an amino acid identical to that of the H120 strain. A *dash* indicates an amino acid deletion in comparison with the H120 strain. The red box indicate the insertions between the position of 87 and 88 and the position of 119 to 120 (numbered by H120)

For the S1 gene, the nt and aa sequence identities between strain GD and other IBV strains were 61.6–98.3 and 49.8–96.8 %, respectively. Isolate GD showed the highest identity to strain TW2575/98 (98.3 % nt and 96.8 % aa sequence identity), which is much higher than other strains. The nt and aa identities of S1 gene of strain GD and H120 vaccine strain did not exceed 82 % (Table 1). There were three insertions in the deduced aa sequence of S1 when compared with strain H120. One was between positions 87 and 88, the other two were between 119 and 120 (H120 aa numbering). The insertion positions were located in or near hypervariable region 1 (HVR1) and HVR2, the regions that had a close relationship with a neutralizing antibody. The insertion of glycine between positions 87 and 88 was also detected in strain TW2575/98 and TW1171/92 which were classified in TW-type but was not detected in other reference strains. We also found that the second insertion of lysine between 119 and 120 was different from the other reference strains except TW-type IBVs (Fig. 1). Furthermore, there were 98 aa mutations and no deletion in the S1 protein of strain GD. Of these 98 mutations, 68 were located in the N-terminal 300 aa that contained HVR1, HVR2, and HVR3.

The nt and aa sequence identity among the other structural and nonstructural genes of S2, 3a, 3b, E, M, 5a, 5b, and N of strain GD and other strains were 74.7–98.0 and 58.1–97.8 %, respectively (Table 1).

### Phylogenetic analysis of IBV sequences

**Fig. 2:**
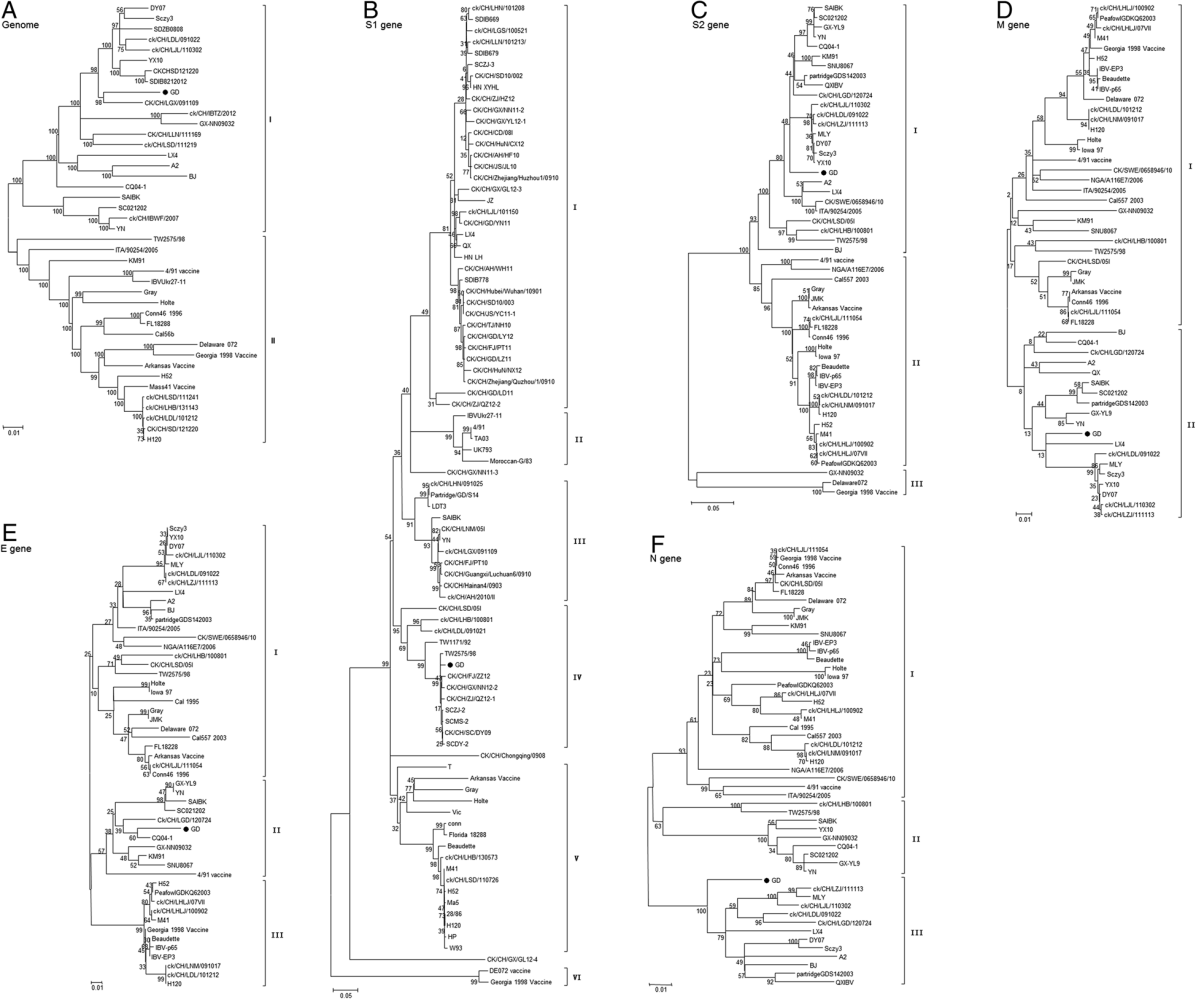
Phylogenetic tree of IBV strains showing the evolutionary relatedness of genomes and individual gene sequences. Complete genome (**a**), S1 gene (**b**), S2gene (**c**), E gene (**d**), M gene (**e**), and N gene (**f**). The neighbor-joining method was used with a bootstrap of 1000 replicates. A black dot (●) indicates strain GD

The phylogenetic tree in Fig. 1 showed the genetic relationships of the full-length sequences of strain GD with a range of other strains representing the majority of known IBV genotypes clustered into two groups (groups I and II). Strain GD belonged to group I and was closely related to most of the prevalent isolates, but distantly related to the Massachusetts-type strains comprising the most common vaccine strains in China (Fig. 2a).

Phylogenetic trees were constructed from the S1 genes of 90 IBV strains prevalent over the last 5 years. The findings indicated that IBVs could be divided into six main clades. Strain GD was located in group IV, also called the TW-type group as it contained the Taiwanese strains, and this group showed low similarity with strains from mainland China. The Massachusetts-type vaccines, such as strain H120, was clustered into group V (Fig. 2b).

Phylogenetic analysis based on the S2, E, M, and N gene sequences suggested that strain GD was closely related phylogenetically to the QX-type or YN-type IBV isolates, which were currently prevalent in China, and distantly related to the Massachusetts-type vaccine strains. So the results showed that GD strain was clustered in different classification based on the phylogenetic analysis of different genes, indicating that recombination events might occur between the S1 gene and other genes (Fig. 2c–f).

### Recombination in the genome of strain GD

As supported by the data from all of the employed recombination detection methods, strain GD was found to undergo potential recombination events in four areas of the genome containing all of the structural genes (Table 2). Both 1ab gene and the S gene showed a high frequency of recombination events. The recombination events mainly occurred between QX- and YN-type IBVs, between QX- and TW-type IBVs, and between QX- and QX-type IBVs.

**Table 2:** Recombination breakpoints, genes, and major and minor related sequences in the genome of strain GD

Breakpoints		Genes	Major sequence	Minor sequence	Detection method
Start	End				
597	5519	1ab	BJ (QX-type)	SAIBK (YN-type)	RDP, Bootscan, GENECONV, MaxChi, Chimaera, SiScan, 3Seq
5507	9056	1ab	CK/CH/LDL/091022 (QX-type)	CK/CH/LJL/110302 (QX-type)	RDP, Bootscan, GENECONV, MaxChi, Chimaera, SiScan, 3Seq
20100	22385	S1, S2	CK/CH/LDL/091022 (QX-type)	TW2575/98 (TW-type)	RDP, Bootscan, GENECONV, MaxChi, Chimaera, SiScan, 3Seq
22386	26161	S2, Gene3, M, Gene5, N	CK/CH/IBTZ/2012 (QX-type)	SC021202 (YN-type)	RDP, Bootscan, GENECONV, MaxChi, Chimaera, 3Seq

Only transferred gene fragments where *p* ≤ 1 × 10^−12^ are included in the table. “Genes” indicates the coding sequences contained within the fragment introduced by recombination. The major sequence is the sequence most closely related to that surrounding the transferred fragment. The minor sequence is most closely related to the transferred fragment in the recombinant

### Analysis of TW-type strains isolated in China

**Fig. 3:**
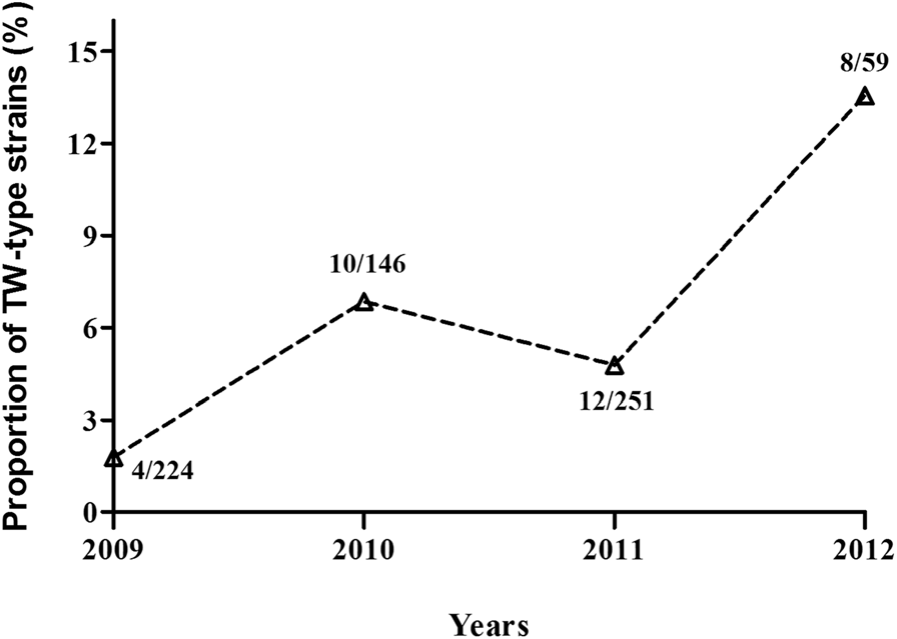
Proportion of TW-type domestic strains in recent years in China. Each denominator (224, 146, 251, 59) represent the numbers of IBV strains during different years, while the numerator (4, 10, 12, 8) represent the numbers of TW-type strains. All the data used were from NCBI

There is no TW-type strains isolated from 2006 to 2008 and few sequence data of IBV S1 gene released from 2013 to 2015. Therefore, the data of TW-type strains available from year 2009 to 2012 were used in our analysis. Based on sequence and phylogenetic analysis, 61 TW-type strains were identified, comprising 20 strains from Taiwan and 41 from mainland China. Interestingly, there were two TW-type strains, strains GX-G and GX-XD, isolated in Guangxi Province in 1988. Another strain, CK/CH/LSD/05I, classified as a TW-type strain, was isolated in Shandong Province in 2005 and first reported in 2008. These results demonstrated an increasing ratio of TW-type strains in mainland China in the period of 2009–2012 (Fig. 3).

## Discussion

Infectious bronchitis has had a devastating effect on the domestic poultry industry since the emergence and increased prevalence of IBV in China. Because of the high rate of mutation and recombination in coronaviruses, new serotypes and genotypes of IBV have been frequently reported in recent years around the world [2]. These new isolates were generated by insertions, point mutations, and recombination, highlighting the importance of genomic sequence analysis of new isolates of IBV [18]. This study was undertaken to investigate the genetic characteristics of a new IBV strain isolated in Guangdong Province of China in 2013. Sequence, phylogenetic, and recombination analysis were performed to explore the genetic features of this new epidemic isolate, alongside analysis of TW-type strains isolated in mainland China.

The S gene of IBV was expressed as precursor protein (S_0_), then cleaved into two subunits S1 and S2. The S1 protein determines the genotype, serotype, and phenotype of IBVs [19]. In this study, the S1 cleavage site of strain GD was R-R-F-R-R (Arg- Arg- Phe- Arg- Arg), which was commonly observed in recent Chinese isolates and was identical to the most of TW-type IBVs isolated in China mainland. However, it has been reported that the cleavage recognition site in the S1 protein was not associated with the pathogenicity of IBV [20, 21]. The S1 gene of strain GD was most closely related to TW-type strains due to the highest identity identified between strain GD and TW2575/98 (isolated in Taiwan). Previous studies suggested that only a few changes in the aa sequence of the S1 protein of IBV could lead to the emergence of a new serotype [22], suggesting that amino acid insertions and mutations in the S1 protein might lead to the changes of immunogenicity in strain GD, which make it possible to escape the immunization of current vaccine. Further investigations are still required.

Phylogenetic analysis of the S1 gene in this study suggested that all strains were clustered into six distinct genetic branches. Group I strains were proposed to be QX-like strains as the Chinese QXIBV strain was involved. QX-like IBV strains have become the predominant genotype in China and other countries in Asia and Europe [23]. Group III strains were designated as YN-type strains due to the YN strain isolated in southern China in 2005 [16]. The group IV strains, known as the TW-type strains because of inclusion of the Taiwanese reference strains, formed a unique genotype that was separated from the Chinese genotypes [24, 25].

An increasing number of recombination events have been reported in IBVs, which occurred between different IBV strains, not only between field isolates but also between field and vaccine viruses [26], causing the emergence and evolution of new IBV genotypes [27, 28]. Previous studies have suggested that recombination within the 1ab, S1, S2, 3a,3b, E, M, and N structural proteins had likely occurred in most parts of the world [1, 15, 16, 29, 30]. In this study, convincing evidence was obtained to suggest that four independent recombination events occurred in strain GD affecting all of the structural genes. The major parent of strain GD was identified as a QX-type IBV, while the minor parents were TW-type and YN-type IBVs, confirming that RNA recombination of IBV included more than two strains and occurred between multiple genes.

Sequence, phylogenetic, and recombination analysis inferred that isolate GD was closely related to TW-type IBV strains. In addition, there was similar background between GD strain and a majority of the TW-type domestic IBVs, such as the similar days of the affected broilers, the morbidity and mortality rate. These results were in agreement with those of previous studies that reported an increasing number of TW-type strains isolated in southern China in recent years [24, 25, 31]. Taiwan is a location-independent island separated by a strait from mainland China, and no livestock trading occurred between these countries. The main reason for the emergence of IBV variants in Taiwan has been proposed to be the migration of birds [32]. Since the first TW-type domestic strain, CK/CH/LSD/05I, was isolated in Shandong Province, an increasing number of TW-type strains have been isolated in mainland China [33], as confirmed by our findings. Most of the TW-type strains were isolated from about 30-days-old broilers while few were from more than 200-days-old layer hens. Moreover, the majority of TW-type strains (thirty-one over forty-one) have been isolated in southern China, with only seven strains isolated in northeast China. Geographical factor is one of the factors affecting the evolution of IBVs. Northeast China is located further from Taiwan island than the southern. That may be the reason we can explain why most of the isolates are from in Southern China. Our findings indicated that mutations and/or recombination events were common among IBVs isolated in China. The increasing ratio of TW-type strains suggested that conventional vaccines such as H120 might not be effective in protecting poultry.

When live-attenuated and inactivated vaccines of IBV were first introduced they were effective at controlling infectious bronchitis among poultry flocks [34]. However, serious production losses in vaccinated flocks have been experienced in many areas of China in recent years [22, 35, 36]. The isolation of IBV strains from vaccinated flocks suggested that IBV strains had evolved to evade the immune defenses induced by current vaccines, highlighting the importance of close monitoring and analysis of emerging strains.

## Conclusion

In summary, our data suggested that both inter-strain recombination and mutation were contributing to the generation of IBV variants in the field. We showed here that genomic recombination between IBV strains might lead to the replacements of large RNA fragments in multiple genes. Sequence and phylogenetic analysis of strain GD revealed that this isolate was likely a recombinant of QX- and TW-type strains. The emergence of new TW-type strains has been increasing in southern China in recent years, and TW-type isolates are genetically distantly related to the predominant vaccine strain H120. Our research therefore emphasizes the significance of continuous monitoring and new vaccine strategy in view of the current circulating strains, which are the fundamental of the prevention and control infectious bronchitis among poultry.

## Methods

### Virus isolation and amplification

A strain of IBV was isolated from 32-day-old broiler chicks from a flock which were vaccinated with an IBV vaccine containing the ‘Massachusetts-type’ serotypes in Guangdong Province of China in 2013. The flock exhibited IB features described as decrease in feed consumption, respiratory symptoms and had a morbidity of 60 % and a mortality of 5 ~ 6 %. The isolate was propagated in the allantoic cavity of 10-day-old embryonated specific-pathogen-free eggs and the embryos were incubated at 37 °C for 40 h. The allantoic fluid was harvested sterilely from the embryos after been passaged three times, and was frozen at −80 °C prior to use.

### PCR primer design

Twenty-eight pairs of primers were designed based on the complete genome sequence of IBV strain TW2575/98 from the GenBank database (GenBank accession number DQ646405) and these primers were used for the amplification of the complete genome of strain GD (Table 3).

**Table 3:** Primers used for complete genome sequence amplification of strain GD

Primer^a^	Location (bp)	Upstream primer	Downstream primer	Length (bp)
1	22–1571	TATATATCTATTGCACTAGCC	AGTCAGACAGACAACACGCT	1549
2	1025–1677	GCAGACTTGTTGGTGAGGTTA	CACAAGTTCCGAAACACTAAA	652
3	1530–2687	TGGAGGGACATCTTTGCTAT	TCTGTCTCAACTTCAATGGG	1157
4	2566–3961	TGGTGAAACTACTGTGAAGG	CACACCATCTACAAGAACAT	1395
5	3642–5168	TGTTAACGCCGCAAATGAG	GGCAACTTGGAATCTTCCT	1527
6	4873–6504	TAAAAAGAGTAAGAGCAAGA	AGGAGACATAAGTGTATTTTG	1631
7	6534–7920	CGTCTACACTAACTCAGGCTA	CCTGACTCCACTAGGTTGAA	1386
8	7890–9260	GAAATTGTTGGTTACACCCAC	TAGAACGCATAGTAACGGGG	1370
9	9247–10664	TCAGTAGGCGTTTGAAAGG	ATAGGCAACACACGGTCA	1417
10	10594–12110	GGCATAGGTTGGATGTTTACT	CACAGAAGCCCCTCCGTAA	1516
11	11954–12523	GTCTTACAGTCTAAAGGAT	AGCACAGTTACGCTTCAAAT	569
12	12418–13418	ACTTAGACAACCAAAACCCTT	TCATAATAACACCGAGTTCCT	1000
13	13261–13978	CCCTCCTCAAGTATGATTAT	ATAGTGGGCAGGACATTCTT	717
14	13865–15044	TATTTTGTTTAGAGGTGACG	AGGAATAGTCAATAAGCAT	1179
15	14712–16138	CTGATTCTAAGTGTTGGGTT	TCCTTTGAGGTACTATGCGA	1427
16	15516–16465	ACTCGCTAAGACGCTTTGCT	TTAGGACAACGGTAACACTTC	950
17	16283–17509	GTGACATTCTATTGGTTGAT	ACAGACTTGTTCCTTGCCT	1226
18	17503–19024	TCACTTGAGAGCTTTGT	TAAACATACAGATTCGCT	1521
19	18628–19634	ATCAAACAACTCTGCCTACA	CCACATTCATCATAATACCACT	1007
20	18945–20442	AAGCGGTATYCNTATGTAGA	ATAGTRCAVACAAAAKRGTCA	1498
21	20189–20578	TTATTTGGGTGACAGTGG	ACCACTAATAATACCAACAG	389
22	20437–22232	AAGGTTAATCCCTGTGAAG	AGTYTCVGTAAGAATAGCA	1795
23	21873–22919	AAGGTTAATCCCTGTGAAG	AGTYTCVGTAAGAATAGCA	1047
24	22723–23837	CTTTTGCHACTCAGATDCA	AGATTTCTTACCACACTTACT	1115
25	23791–24992	AGGGGCTTAATGACTCTCTT	CTGACCTTCACAATAAAGAAC	1201
26	24796–25894	GCAGCGATAATACTTACAGT	TCTGCTTGTCCTGCTTTGT	1099
27	25121–26453	GTGACCGAAGCGGAAATAA	TCAGAGGAATGAAGTCCCAAC	1333
28	26891–27706	GGTGATTCTCAAGATGGTAT	GCTCTAACTCTATACTAGCCT	1416

^a^Primer locations are indicated according to strain TW2575/98 (DQ646405)

### Viral RNA extraction, reverse transcription (RT)-polymerase chain reaction (PCR), and DNA sequencing

Viral RNA was extracted from the virus-infected allantoic fluid using TRIzol reagent (Invitrogen, Carlsbad, CA, USA) following the manufacturer’s instructions. RT was carried out using 4 μL of total RNA, 2 μL of dNTPs (2.5 mM), 1 μL of RNasin (50 U/μL), and M-MLV (10 U/μL, Promega, Madison, WI, USA) at 37 °C for 1 h, and 95 °C for 5 min. For PCR, 25 μL of 2 × PCRmix (TransGen Biotech, Beijing, China) and 20 pmol of each primer were added to 100 ng of template cDNA in a total reaction volume of 50 μL. The reaction conditions were as follows: 94 °C for 5 min followed by 30 cycles of 94 °C for 45 s, annealing for 45 s (temperature depended on the primers), 72 °C for 2 min, with a final extension step of 72 °C for 10 min. The PCR products were analyzed by 1 % agarose gel electrophoresis. Amplified sequences were purified using the AxyPrep DNA Gel Extraction kit (Axygen, Union City, CA, USA), and the purified products were inserted into the pMD18-T vector (Promega). Nucleotide sequencing of the recombinant plasmids was performed by TsingKe Biological Technology (Beijing, China).

Every passages of the virus-infected allantoic fluid were sequenced according to the method above and the last passage was sequenced for three times to ensure the accuracy of the sequence of GD strain.

### Sequence and phylogenetic analysis

Assembly of the sequence of the IBV GD isolate was conducted using the SeqMan program of the DNASTAR software suite version 7.1 (DNASTAR, Madison, WI, USA). The sequence of the full-length genome and the individual structural genes were compared with other IBVs using the MegAlign program of the DNASTAR software. Deduced amino acid (aa) sequences were aligned and phylogenetic trees were mapped with the full-length genomes and structural genes using MEGA 5.0 software (www.megasoftware.net) and the Kimura 2-parameter nucleotide substitution model. The maximum likelihood method was used with 1000 bootstrap replicates.

### Recombination detection

The complete genome sequence of IBV strain GD and other reference strains were aligned using the ClustaW program of the MEGA 5.0 software and were analyzed using the Recombination Detection Program version 4.0 (RDP4.0, Simmonics, University of Warwick, Coventry, UK) and the algorithms of RDP, Bootscan, GENECONV, MaxChi, Chimaera, SiScan, and 3Seq. Default settings were used for all programs. More than one method was used to analyze the data because results from only a single method were not reliable and could have resulted in misleading results.

### Analysis of TW-type strains isolated in China

Using the data released from NCBI, all S1 gene sequences from IBVs deposited up until November 2015 were collected and phylogenetic analysis was performed. The proportion of TW-type strains among the isolates were computed for each year.
